# Modelling the relationship between malaria prevalence as a measure of transmission and mortality across age groups

**DOI:** 10.1186/s12936-019-2869-9

**Published:** 2019-07-23

**Authors:** Sammy Khagayi, Meghna Desai, Nyaguara Amek, Vincent Were, Eric Donald Onyango, Christopher Odero, Kephas Otieno, Godfrey Bigogo, Stephen Munga, Frank Odhiambo, Mary J. Hamel, Simon Kariuki, Aaron M. Samuels, Laurence Slutsker, John Gimnig, Penelope Vounatsou

**Affiliations:** 1 Kenya Medical Research Institute-Center for Global Health Research, Kisumu, Kenya; 2 Swiss Tropical and Public Health Institute, Basel, Switzerland; 3 University of Basel, Basel, Switzerland; 4 Malaria Branch, Division of Parasitic Diseases and Malaria, Center for Global Health, Centers for Disease Control and Prevention, Atlanta, GA USA; 5 Centers for Disease Control and Prevention, Kisumu, Kenya

**Keywords:** Malaria, Mortality, Parasite prevalence, Bayesian spatio-temporal, Health and demographic surveillance system

## Abstract

**Background:**

Parasite prevalence has been used widely as a measure of malaria transmission, especially in malaria endemic areas. However, its contribution and relationship to malaria mortality across different age groups has not been well investigated. Previous studies in a health and demographic surveillance systems (HDSS) platform in western Kenya quantified the contribution of incidence and entomological inoculation rates (EIR) to mortality. The study assessed the relationship between outcomes of malaria parasitaemia surveys and mortality across age groups.

**Methods:**

Parasitological data from annual cross-sectional surveys from the Kisumu HDSS between 2007 and 2015 were used to determine malaria parasite prevalence (PP) and clinical malaria (parasites plus reported fever within 24 h or temperature above 37.5 °C). Household surveys and verbal autopsy (VA) were used to obtain data on all-cause and malaria-specific mortality. Bayesian negative binomial geo-statistical regression models were used to investigate the association of PP/clinical malaria with mortality across different age groups. Estimates based on yearly data were compared with those from aggregated data over 4 to 5-year periods, which is the typical period that mortality data are available from national demographic and health surveys.

**Results:**

Using 5-year aggregated data, associations were established between parasite prevalence and malaria-specific mortality in the whole population (RR_malaria_ = 1.66; 95% Bayesian Credible Intervals: 1.07–2.54) and children 1–4 years (RR_malaria_ = 2.29; 1.17–4.29). While clinical malaria was associated with both all-cause and malaria-specific mortality in combined ages (RR_all-cause_ = 1.32; 1.01–1.74); (RR_malaria_ = 2.50; 1.27–4.81), children 1–4 years (RR_all-cause_ = 1.89; 1.00–3.51); (RR_malaria_ = 3.37; 1.23–8.93) and in older children 5–14 years (RR_all-cause_ = 3.94; 1.34–11.10); (RR_malaria_ = 7.56; 1.20–39.54), no association was found among neonates, adults (15–59 years) and the elderly (60+ years). Distance to health facilities, socioeconomic status, elevation and survey year were important factors for all-cause and malaria-specific mortality.

**Conclusion:**

Malaria parasitaemia from cross-sectional surveys was associated with mortality across age groups over 4 to 5 year periods with clinical malaria more strongly associated with mortality than parasite prevalence. This effect was stronger in children 5–14 years compared to other age-groups. Further analyses of data from other HDSS sites or similar platforms would be useful in investigating the relationship between malaria and mortality across different endemicity levels.

**Electronic supplementary material:**

The online version of this article (10.1186/s12936-019-2869-9) contains supplementary material, which is available to authorized users.

## Background

There has been a substantial reduction in malaria related mortality worldwide over the last decade, however, the burden is still disproportionately felt in sub-Saharan Africa (SSA) [1]. Due to the high burden in children and pregnant women [2], malaria control intervention resources in previous years have been targeted to these vulnerable populations. Increased quality data on malaria infection dynamics and mortality across all ages [3] has created an increased awareness of the burden of disease amongst the other populations, and policies have been expanded to ensure universal coverage with effective vector control methods (e.g. long-lasting insecticidal nets [LLIN]), availability of diagnostics (e.g. rapid diagnostic tests, RDT), and availability of appropriate treatments (e.g. artemisinin-based combination therapy, ACT) to all.

There is evidence that the malaria burden in older children and adults in terms of mortality and parasite prevalence [4–6] is higher than had been thought of previously. With data analysed from a health and demographic surveillance system (HDSS) in western Kenya run by the Kenya Medical Research Institute (KEMRI) and Centers for Disease Control and Prevention (CDC) showing that, largely due to increased malaria/HIV prevention and treatment interventions, malaria mortality rates decreased in young children and persons aged ≥ 15 years, but remained stable in 5–14 year olds [6]; suggesting that malaria control efforts should be intensified in this group. Furthermore, older children and adults have been shown to act as reservoirs of transmission due to high levels of asymptomatic infections [7], supporting the current policy of universal coverage of malaria control interventions.

Measuring malaria transmission intensity and its effect on mortality can be used to monitor disease burden and assess the impact of interventions and control programmes. This has been done previously using entomological inoculation rates (EIR) [8, 9]; however, measuring EIR is expensive, time consuming and is often imprecise, particularly in low transmission settings. Other measures of malaria transmission include slide positivity rate (SPR), parasite prevalence, disease incidence, sporozoite rate, and vectorial capacity [10–13].

Malaria parasite prevalence (PP) surveys carried out mostly during peak transmission times through representative sampling of populations are a preferred method for measuring malaria burden because reporting from weak or non-existent health systems is inadequate to measure incidence [14], at the same time health facilities do not capture asymptomatic infections which are important for malaria transmission. Furthermore, PP survey data are easier to interpret and less prone to uncertainty compared to other measures [15]. These surveys are however limited in their ability to capture malaria morbidity, seasonality of transmission and monitor temporal trends from surveys that are not seasonally aligned [16]. With regular, consistent survey intervals, stringent methodology in sampling and diagnosis, PP surveys can provide measures of malaria transmission which are useful to policy makers.

Due to their nature, HDSS sites can be used to collect data that are well aligned in space and time, so as to investigate variations in malaria transmission in relation to morbidity and mortality. They provide data on mortality across age groups, and in conjunction with PP surveys, offer a unique platform through which the relationship between malaria transmission and mortality can be investigated while taking into consideration spatio-temporal factors [16–18] and hence monitor the impact of interventions over time. One such project, the Malaria Transmission Intensity and Mortality Burden across Africa (MTIMBA) investigated the effect of EIR as a measure of exposure and its effect on mortality in several HDSS sites in Africa and showed that small changes in transmission dynamics as measured by EIR, impact greatly on mortality [8, 9].

This study sought to understand how malaria parasite prevalence and clinical malaria translate into mortality and consequently help inform national control programmes on how to best use their survey data in estimating mortality. The relation between malaria prevalence and mortality was explored across all age-groups using Bayesian geostatistical models on data collected between 2007 and 2015 from the KEMRI and CDC HDSS (KHDSS) site in western Kenya. There have been no studies done to investigate the usefulness of PP and its association with mortality using data that are well aligned in space and time across different age groups in similar settings; hence enriching the knowledge of malaria transmission-mortality using high quality consistent data. Since estimation of malaria deaths is still not very clear, results of this study would clarify the potentials of PP surveys to reliably estimate malaria deaths and as its intensity reduces worldwide, help inform decisions on resource allocation and monitoring the impact of interventions.

## Methods

### Study area and population

The KHDSS located in Siaya County of western Kenya follows a population of over 240,000 people as of mid-2015 in an area of over 700 km^2^ [19]. This HDSS is located in a malaria endemic zone with a high burden of HIV/AIDS compared to the rest of the country [20, 21].

From the HDSS, data on an initial population at baseline was collected followed by subsequent 4 monthly cycles every year during which data were collected on births, deaths, in-migration and out-migrations. These data were used to estimate person-years of observation (pyo) that served as a denominator to calculate mortality rates. Verbal autopsy (VA) was used to determine malaria-specific mortality rates. The methods used for verbal autopsy have been described in detail elsewhere [19, 22]; it involves capturing data on a deceased person’s last illness, signs, symptoms and medical history which is then used to determine the most probable cause of death using a computer-based Bayesian expert algorithm called InterVA [23].

### Malaria prevalence

Annual all-age malaria and anaemia prevalence surveys were conducted by randomly sampling compounds within the HDSS, and testing all consenting members of the compound for malaria by blood smear microscopy, from the population during the peak malaria transmission period in July. Details of the sampling by year are shown in Additional file 1. Trained interviewers then visited the compounds, administered a questionnaire to collect information on demographics, risk factors for malaria infection, healthcare-seeking, previous illness, socioeconomic status, LLIN ownership/use, and collected a blood sample to prepare thick and thin smears for microscopy. The blood slides were transported to a central laboratory, stained with 10% Giemsa and examined for malaria parasites by expert microscopists.

Two measures of transmission were considered; prevalence of malaria parasites and clinical malaria for comparative purposes. Parasite prevalence by age group, village, and year was defined as the proportion of participants in each village that had malaria by microscopy out of all the participants from the same village who were tested for malaria. Similarly, clinical malaria prevalence was defined as the proportion of participants in each village who had malaria parasites of any density by microscopy in combination with either a reported fever in the previous 24 h or a temperature of 37.5 °C and above out of all those tested.

### Data management and statistical analysis

Rates of clinical malaria and PP were aggregated at village level and linked to mortality data by village, year of study and age group. The age groups were defined as: 0–28 days (neonates), 1–11 months (infants), 1–4 years (child), 5–14 years (older child), 15–59 (adults) and 60+ (elderly). Crude and age specific all-cause/malaria-specific mortality rates were calculated by dividing the deaths in each group with the total person-years observed (pyo) in that group.

A measure of socioeconomic status was constructed based on household asset ownership using a composite score, derived from multiple correspondence analysis (MCA) [24] and categorized into 3 levels as least poor for the well off, poor for the average and poorest for the lowest rank while LLIN coverage was calculated as the percentage of households in a village owning at least one net per two people in a given year. Distance to health facilities was calculated as the networked distance of each household from the nearest health facility, and classified into 3 categories as less than 1 km, 1 to 2 km and greater than 2 km. the elevation of each household was downloaded from the remote sensing United States geological survey (USGS) Earth Resources Observation and Science (EROS) website [25]. These variables were also aggregated at village level and linked to the parasitaemia and mortality data.

The analysis considered two approaches; in one approach, the data were aggregated on a yearly basis, hence 9 years of observation; the second approach was aggregating the data into two periods (2007–2010 and 2011–2015).

For each age group, Bayesian negative binomial geostatistical models were fitted to assess the relationship between PP and all-cause/malaria-specific mortality. Variable selection based on bivariate negative binomial models was used to identify potential confounders. Variables with a p-value below 0.1 were included in the final geostatistical models so as not leave out important variable whose effects would be missed when investigated alone, but become important if included in combination with other factors. Spatial correlation was taken into account by village specific random effects modelled via a Gaussian process with a mean of zero and an exponential correlation matrix of the distance between villages in the study [26]. Bayesian models were fitted in OpenBugs version 3.1.2 (Imperial College and Medical Research Council London, UK) using Markov Chain Monte Carlo (MCMC) simulation for parameter estimation. Regression coefficients from the Bayesian geostatistical model were exponentiated to obtain prevalence rate ratios (PRR) and summarized by their posterior median and 95% Bayesian Credible Intervals (BCI). Covariate effects were considered statistically important when the BCI of the corresponding regression coefficients on the log scale did not include zero. Due to the nature of Bayesian statistical inference, the terminology of statistically significant was replaced by statistically important effect when reporting results. In this paper, the results for the association between clinical malaria and all-cause mortality are presented; clinical malaria and malaria-specific mortality; and lastly PP and both all-cause/malaria-specific mortality in that order. Model formulation details are provided in Additional file 2.

## Results

### Descriptive statistics

Between the year 2007 and 2015, over 441,000 individuals were enrolled/monitored in the HDSS contributing a total of 2,114,223 pyo and 26,283 deaths, for an average crude death rate of 12.4 (95% confidence interval; 12.3–12.6) deaths per 1000 pyo as shown in Table 1.

**Table 1:** All-cause/malaria specific mortality, clinical malaria and malaria parasite prevalence by year

Year	Person years of observation	Sampled population	Malaria parasite prevalence	Clinical malaria prevalence	All-cause mortality rate per 1000 pyo	Malaria-specific mortality rate per 1000 pyo
2007	181,537	1270	29.6% (27.1–32.2)	7.2% (5.8–8.7)	15.5 (14.9–16.1)	1.3 (1.2–1.5)
2008	230,374	1039	27.3% (24.6–30.2)	6.0% (4.6–7.6)	18.8 (18.2–19.3)	3.5 (3.3–3.7)
2009	230,373	2508	39.0% (37.1–40.9)	7.5% (6.5–8.6)	15.6 (15.1–16.2)	2.9 (2.6–3.1)
2010	233,871	5243	39.7% (38.4–41.0)	7.9% (7.2–8.6)	12.4 (11.9–12.8)	2.1 (1.9–2.3)
2011	238,524	2091	39.2% (37.1–41.3)	8.5% (7.3–9.7)	10.9 (10.5–11.3)	1.4 (1.2–1.5)
2012	246,254	2719	34.1% (32.3–35.9)	7.8% (6.8–8.8)	10.2 (9.8–10.6)	1.4 (1.2–1.5)
2013	249,757	2358	34.5% (32.6–36.5)	10.5% (9.3–11.8)	10.5 (10.1–10.9)	1.6 (1.4–1.7)
2014	252,173	1934	35.6% (33.4–37.8)	8.4% (7.2–9.7)	10.2 (9.8–10.6)	1.0 (0.9–1.2)
2015	251,360	1756	29.8% (27.7–32.0)	7.5% (6.2–9.1)	9.4 (9.0–9.8)	0.9 (0.7–1.0)
Overall	2,114,223	20,918	35.8% (35.2–36.5)	8.1% (7.7–8.4)	12.4 (12.3–12.6)	1.8 (1.7–1.9)

*pyo* person years of observation

**Fig. 1:**
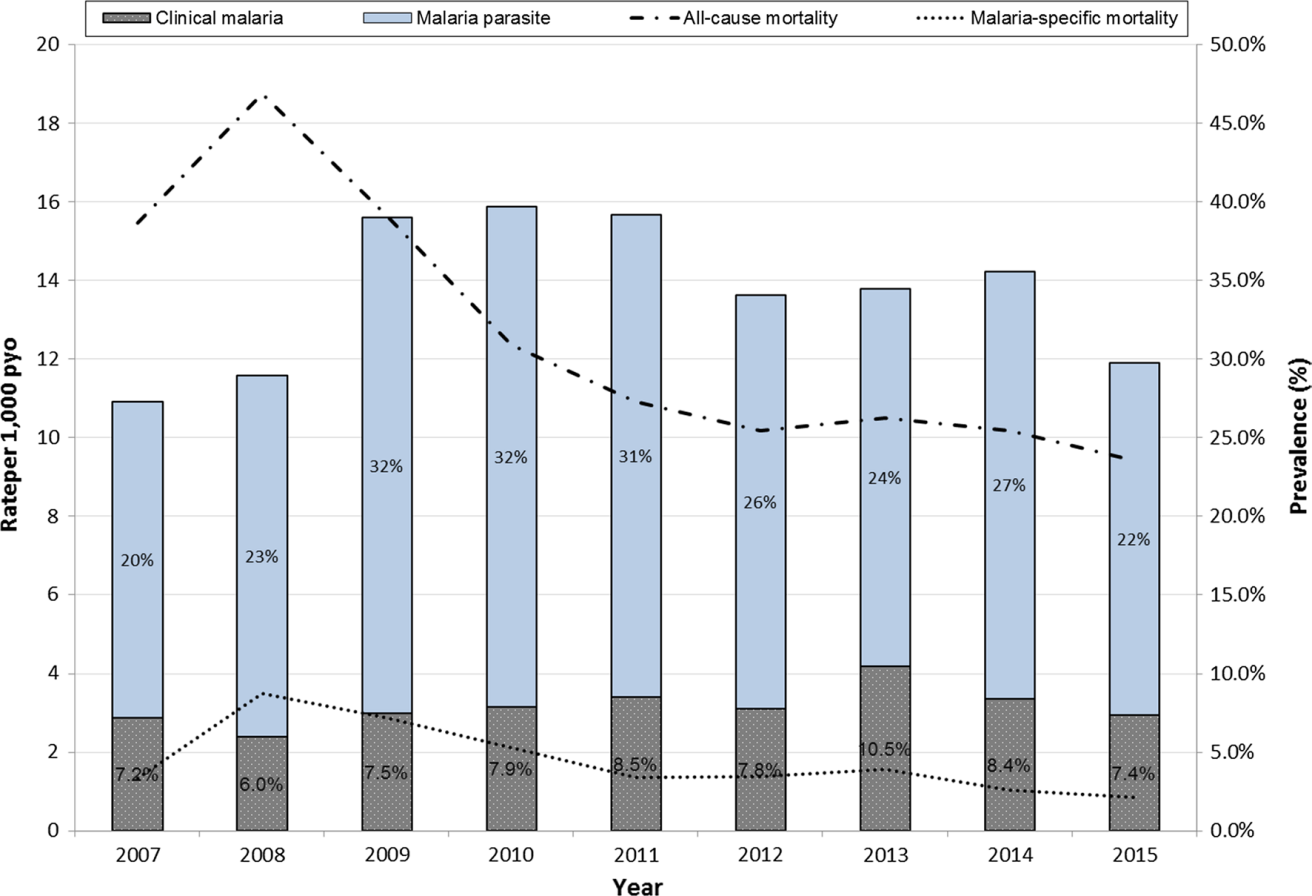
All-cause and malaria specific mortality rates versus malaria parasite and clinical malaria prevalence

All-cause mortality during the study period rose from 15.5 (14.9–16.1) deaths per 1000 pyo in 2007 to 18.8 (18.2–19.3) in 2008 then dropped to a low of 9.4 (9.0–9.8) in the year 2015 with malaria-specific mortality following a similar trend; rising from 1.3 (1.2–1.5) deaths per 1000 pyo in 2007 to a high of 3.5 (3.3–3.7) in 2008, but eventually dropping to 0.9 (0.7–1.0) deaths per 1000 pyo in 2015 (Table 1). The average PP during the whole study period was 35.8% (35.2–36.5); ranging between 27.3% in 2008 to a high of 39.7% in 2010 but then dropped over the years to 29.8% in 2015. A further breakdown of the positive slides showed that 8.1% (7.7–10.4) of the respondents had clinical malaria (parasites and fever); on average one fifth of all the positives had clinical malaria (Fig. 1).

**Fig. 2:**
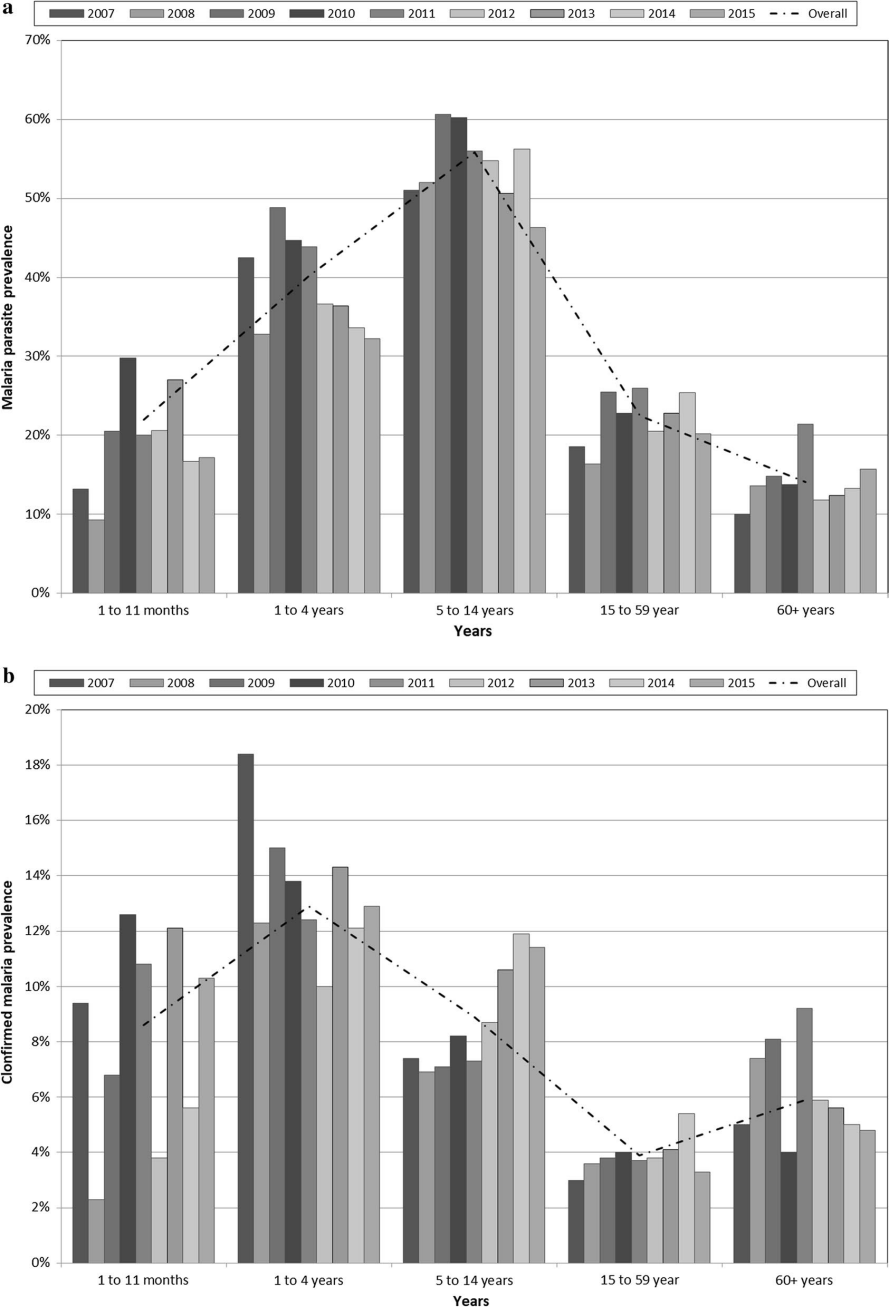
Malaria parasite prevalence (**a**) and clinical malaria (**b**) by age groups

The highest parasite prevalence was observed among older children aged 5–14 years, with an average PP of 56% (95% CI 54–57), followed by children aged 1–4 years at 40% (39–41), adults at 22% (21–24), and infants at 22% (19–25); the elderly at 14% (12–16) had the lowest rate. The age distribution of prevalence indicates an increase in parasite prevalence from infanthood to older children followed by a drop as the population ages (Fig. 2a). However, by including the presence of fever, a rise in clinical malaria from infants to children aged 1 to 4 years was observed, after which it drops in the 5–14 age-group and in adults but rises slightly among the elderly. The highest prevalence of clinical malaria was in infants with a peak of 18.4% among those tested in the year 2007 (Fig. 2b).

### Model-based results

#### Relationship between clinical malaria and all-cause mortality

The following variables met the criteria for inclusion in the age-specific geostatistical mortality models: reported net usage, distance to health facilities, socioeconomic status, year of study and altitude. For comparability, these variables were included in the Bayesian models fitted by age group. Results in Table 2 show that the prevalence of confirmed malaria when aggregated over four and 5-year periods, was associated with all-cause mortality in the combined age groups (RR = 1.32; 95% BCI: 1.01–1.74), in the 1–4 year olds (RR = 1.89; 1.00–3.51) and in the 5–14 year olds (RR = 3.94; 1.34–11.1). Increase in distance to health facilities was associated with higher mortality among neonates, children aged 1–4 years and the combined age group analysis. Risk of all-cause mortality was higher in the period 2007–2010 compared to 2011–2015 in all ages except in neonates. Higher SES and increased elevation were both associated with lower mortality. The association between reported net use and mortality was not statistically important across most age groups save for the elderly. The minimum distance at which spatial correlation was below 5% ranged from 13.2 km to 50 km for all the age groups. The analyses of the yearly prevalence data did not show a statistically important relation between confirmed malaria and all-cause mortality (see Additional file 3).

**Table 2:** Posterior estimates showing effects of prevalence (PP and confirmed malaria) on all-cause mortality

Covariate	Neonates	Infants	1–4 years	5–14 years	15–59 years	60+	Overall^b^
	RR (95% BCI^a^)	RR (95% BCI)	RR (95% BCI)	RR (95% BCI)	RR (95% BCI)	RR (95% BCI)	RR (95% BCI)
PP^c^	0.64 (0.29–1.34)	1.32 (0.86–2.04)	1.50 (0.98–2.23)	1.38 (0.69–2.69)	1.23 (0.94–1.57)	1.04 (0.78–1.35)	1.15 (0.96–1.37)
Clinical malaria^c^	1.05 (0.30–3.51)	1.65 (0.81–3.26)	1.89 (1.00–3.51)	3.94 (1.34–11.1)	0.99 (0.64–1.54)	1.07 (0.69–1.66)	1.32 (1.01–1.74)
Net use	1.18 (0.70–2.03)	1.06 (0.80–1.44)	0.82 (0.63–1.08)	0.97 (0.60–1.59)	0.88 (0.73–1.06)	0.78 (0.64–0.94)	0.91 (0.81–1.02)
Distance to health facility							
0–1 km	1	1	1	1	1	1	1
1–2 km	1.05 (0.81–1.37)	1.04 (0.90–1.20)	1.06 (0.93–1.22)	1.01 (0.80–1.28)	1.08 (0.99–1.18)	0.03 (0.94–1.13)	1.07 (1.01–1.14)
> 2 km	1.33 (1.01–1.76)	1.06 (0.91–1.24)	1.16 (1.00–1.34)	1.28 (0.99–1.65)	1.09 (0.99–1.20)	1.07 (0.97–1.18)	1.12 (1.05–1.20)
SES							
Poorest	1	1	1	1	1	1	1
Poor	1.01 (0.78–1.29)	0.93 (0.80–1.07)	0.94 (0.82–1.07)	0.86 (0.68–1.08)	0.91 (0.83–0.99)	0.97 (0.88–1.06)	0.95 (0.90–1.01)
Least poor	0.83 (0.63–1.07)	0.85 (0.74–0.98)	0.84 (0.73–0.96)	0.91 (0.73–1.13)	0.88 (0.81–0.97)	0.95 (0.87–1.04)	0.89 (0.83–0.95)
Period							
2007–2010	1	1	1	1	1	1	1
2011–2015	1.05 (0.85–1.30)	0.57 (0.51–0.64)	0.60 (0.54–0.68)	0.77 (0.64–0.93)	0.68 (0.63–0.73)	0.96 (0.89–1.04)	0.71 (0.68–0.75)
Elevation							
1147–1243	1	1	1	1	1	1	1
1244–1293	1.13 (0.80–1.59)	1.09 (0.88–1.34)	1.15 (0.94–1.42)	0.81 (0.59–1.01)	1.03 (0.89–1.18)	0.98 (0.86–1.10)	1.01 (0.89–1.12)
1294–1327	0.84 (0.57–1.23)	0.80 (0.62–1.03)	0.97 (0.78–1.24)	0.98 (0.71–1.36)	0.99 (0.85–1.15)	0.92 (0.80–1.05)	0.87 (0.76–0.99)
1328–1365	1.17 (0.79–1.73)	0.82 (0.63–1.07)	0.99 (0.78–1.27)	0.96 (0.67–1.39)	0.93 (0.78–1.10)	0.96 (0.83–1.11)	0.89 (0.75–1.01)
> 1365	0.92 (0.59–1.44)	0.61 (0.45–0.81)	0.80 (0.62–1.06)	1.09 (0.74–1.63)	0.92 (0.75–1.12)	0.98 (0.84–1.15)	0.80 (0.68–0.93)
Spatial variance	0.49 (0.17–2.74)	0.86 (0.25–4.11)	0.77 (0.23–4.03)	0.51 (0.18–2.02)	0.57 (0.14–2.27)	0.34 (0.15–0.97)	0.96 (0.32–3.69)
Range^d^	24.07 (8.41–90.88)	15.25 (8.22–83.71)	17.38 (8.27–81.66)	23.59 (8.43–88.54)	19.77 (8.34–93.87)	50.09 (11.87–96.65)	13.23 (8.17–51.33)

Mortality and malaria data aggregated by 4 to 5-year periods (i.e. 2007–2010 and 2011–2015)

^a^The effects are presented as the median of mortality rate ratios (RR) and 95% Bayesian Credible Intervals (BCI) adjusted for geographical variation and other predictors

^b^Age-adjusted

^c^They are obtained from different models. Estimates of the rest of the predictors are from the models with confirmed malaria and do not differ from the PP model. PP estimates are only provided for comparison purposes

^d^Minimum distance in kilometres at which spatial correlation is less than 5%

#### Relationship between clinical malaria and malaria-specific mortality

The pattern of association between clinical malaria and malaria-specific mortality across all age groups was similar to that of clinical malaria and all-cause mortality, however, the magnitude of the estimates was higher. The effect of clinical malaria risk on malaria-specific mortality was statistically important and strong among children 5–14 years (RR = 7.56; 1.20–39.54) and 1–4 year olds (RR = 3.37; 1.23–8.93). Meanwhile in the overall population, malaria-mortality rate increases two and half times for every increase in the proportion of clinical malaria by 1% (RR = 2.50; 1.27–4.81) as shown in Table 3. Similar to all-cause mortality analysis, statistically important variables were elevation, distance to health facilities, year of study and socioeconomic status. Reported net use was not statistically important for malaria-specific mortality in any ages except among the elderly (RR = 2.05; 1.04–4.34) in the yearly analysis, where an elevated risk with higher levels of net use was observed. The minimum distance at which spatial correlation was not important (< 5%) ranged from 13.4 to 50.42 km. The analyses of the yearly aggregated data did not show a statistically important relation between confirmed malaria risk and malaria-specific mortality (see Additional file 4).

**Table 3:** Posterior estimates for the effects of prevalence (PP and confirmed malaria) on malaria-specific mortality

Covariate	Infants	1–4 years	5–14 years	15–59 years	60+	Overall^b^
	RR (95% BCI^a^)	RR (95% BCI)	RR (95% BCI)	RR (95% BCI)	RR (95% BCI)	RR (95% BCI)
PP^c^	1.73 (0.74–4.21)	2.29 (1.17–4.29)	0.56 (0.14–2.03)	1.55 (0.48–4.86)	2.24 (0.67–7.44)	1.66 (1.07–2.54)
Clinical malaria^c^	2.23 (0.55–8.36)	3.37 (1.23–8.93)	7.56 (1.20–39.54)	0.60 (0.08–3.91)	0.77 (0.09–5.64)	2.50 (1.27–4.81)
Net use	1.11 (0.61–1.97)	0.74 (0.48–1.14)	1.02 (0.40–2.35)	0.64 (0.28–1.35)	0.72 (0.30–1.77)	0.81 (0.61–1.11)
Distance to facility						
0–1 km	1	1	1	1	1	1
1–2 km	1.11 (0.85–1.48)	0.89 (0.72–1.11)	1.17 (0.75–1.86)	1.36 (0.93–2.03)	1.10 (0.71–1.72)	1.02 (0.89–1.17)
> 2 km	1.19 (0.88–1.61)	1.00 (0.80–1.27)	1.56 (0.97–2.52)	1.13 (0.73–1.76)	1.15 (0.72–1.87)	1.09 (0.94–1.27)
SES						
Poorest	1	1	1	1	1	1
Poor	0.90 (0.67–1.19)	0.83 (0.67–1.03)	1.10 (0.72–1.65)	0.92 (0.61–1.35)	1.08 (0.70–1.63)	0.92 (0.80–1.06)
Least poor	0.96 (0.73–1.26)	0.78 (0.63–0.98)	0.94 (0.62–1.44)	0.86 (0.59–1.25)	1.05 (0.68–1.61)	0.88 (0.76–1.03)
Period						
2007–2010	1	1	1	1	1	1
2011–2015	0.58 (0.45–0.72)	0.56 (0.47–0.67)	0.72 (0.51–1.03)	0.64 (0.47–0.88)	0.68 (0.47–0.96)	0.56 (0.50–0.63)
Elevation						
1147–1243	1	1	1	1	1	1
1244–1293	1.05 (0.75–1.46)	1.17 (0.85–1.61)	0.94 (0.54–1.60)	1.05 (0.62–1.77)	0.89 (0.50–1.55)	1.08 (0.87–1.34)
1294–1327	0.47 (0.31–0.70)	1.10 (0.78–1.58)	1.02 (0.56–1.79)	0.79 (0.45–1.41)	1.00 (0.55–1.75)	0.84 (0.67–1.08)
1328–1365	0.65 (0.43–0.98)	1.00 (0.70–1.47)	0.99 (0.51–1.88)	0.89 (0.49–1.65)	1.15 (0.60–2.14)	0.88 (0.68–1.13)
> 1365	0.36 (0.21–0.58)	0.88 (0.58–1.34)	1.39 (0.69–2.76)	0.58 (0.28–1.16)	1.29 (0.65–2.50)	0.71 (0.52–0.94)
Spatial variance	0.53 (0.17–2.43)	0.75 (0.21–4.40)	0.49 (0.16–2.43)	0.78 (0.18–4.08)	0.64 (0.22–2.65)	0.95 (0.21–7.62)
Range^d^	22.40 (8.42–92.52)	16.00 (8.22–74.12)	28.82 (8.47–91.99)	15.40 (8.21–86.65)	16.87 (8.32–75.36)	15.27 (8.19–78.32)

Mortality and malaria data aggregated by 4 to 5-year periods (i.e. 2007–2010 and 2011–2015)

^a^The effects are presented as the median of mortality rate ratios (RR) and 95% Bayesian Credible Intervals (BCI) adjusted for geographical variation and other predictors

^b^Age-adjusted

^c^They are obtained from different models. Estimates of the rest of the predictors are from the models with confirmed malaria and do not differ from the PP model. PP estimates are only provided for comparison purposes

^d^Minimum distance in kilometres at which spatial correlation is less than 5%

#### Relationship between parasite prevalence and all-cause/malaria specific mortality

The relation between PP and all-cause mortality was not statistically important across all ages (Table 2). However, there was a statistically important association between PP and malaria-specific mortality (Table 3) among children aged 1–4 years (RR = 2.29; 95% BCI: 1.17–4.29), and in the combined age group (RR = 1.66; 95% BCI: 1.07–2.54) when data was aggregated over 5 to 4 year. Analyses of yearly data did not reveal statistically important associations except between PP and all-cause mortality among the adults (RR = 1.23; 95% BCI: 1.01–1.50) (see Additional file 3) and with malaria-specific mortality among the elderly (RR = 3.42; 95% CI: 1.39–8.63) (see Additional file 4).

## Discussion

Using data from community level cross-sectional surveys, it was shown that; parasite prevalence is associated with malaria-mortality in the overall population, while clinical malaria is associated with both all-cause and malaria-specific mortality more so in the age groups 1–4 years and 5–14 years. This relationship was established by fitting over 50 different Bayesian geo-statistical models across different age groups on large data from verbal autopsies, longitudinal household surveys, and cross-sectional malaria parasitaemia surveys carried out annually over 9 years in the HDSS located in a malaria endemic region of western Kenya. These data aggregated over four to 5-year periods showed statistically important relations between clinical malaria and mortality (all-cause and malaria-specific) in the overall population, in children 1–4, and older children aged 5–14 years old, while PP had a statistically important association with malaria-specific mortality in 1–4 year olds and in the overall population. Meanwhile, analyses of the same data, annually aggregated did not establish any association between prevalence of clinical malaria nor PP with either all-cause or malaria-specific mortality across most age groups except for all-cause mortality in adults aged 15–59 years and malaria-specific mortality in the elderly.

Studies in malaria-endemic areas have also shown that children above the age of 5 years are least affected by the malaria burden in terms of confirmed symptomatic malaria and mortality compared to other age groups, even though they remain the biggest reservoir of the malaria parasites [5, 27]. However, the long-term effects of declining transmission on mortality in this age group have not been well explored. This study showed a sevenfold increase in malaria-specific mortality for every 1% increase in clinical malaria prevalence, which was more than twice the effect in children 1–4 year old. This finding could be attributed to low utilization of ITNs by older children compared to other age groups in this study, as well as from previous studies [6] as well as poor health care-seeking behaviour among the same age group [28], resulting in higher mortality rates when data is captured at household level compared to sentinel health facilities. This reinforces the importance of universal coverage of malaria control interventions particularly in high transmission areas.

The absence of an association between PP and all-cause mortality could be due to several factors. First, parasite prevalence from the community might capture more asymptomatic carriers who have acquired immunity from malaria disease, eventually recover without adverse outcomes and hence survive. Second, malaria mortality is usually preceded by severe illness and, therefore, the PP data may be biased, as most of those who were severely ill may have gone to the hospital or succumbed to the disease prior to the time of the survey. Furthermore, an increase or decrease in mortality could be also due to other unmeasured factors that are unrelated to parasite prevalence; an example was shown by the influence of political instability on mortality in the year 2008 in Kisumu [29] that resulted in massive disruption of health delivery.

The lack of association between PP or clinical malaria and mortality in the 15–49 age groups may be an indicator of misclassification of malaria as a cause of death by verbal autopsy. This weakness of verbal autopsy in identifying malaria as a cause of death among adults [30] could result in fewer deaths being classified as malaria than there really are in the population. Evidence suggests that people with HIV have more frequent episodes of symptomatic malaria [31] and that malaria increases HIV plasma viral load and decreases CD4+ T cells [32]. Therefore, an alternative explanation could be that malaria specific mortality among adults may be classified by verbal autopsy as HIV/AIDS-related rather than malaria related.

The estimated effects of PP and clinical malaria were higher for malaria-specific mortality compared to all-cause. Furthermore, clinical malaria was a better predictor of mortality than PP. In fact, some of the asymptomatic infections may neither lead to severe disease nor death and therefore prevalence of clinical malaria is a better indicator for monitoring the disease burden at the population level. The stronger effect of clinical malaria and PP on malaria-specific mortality compared to all-cause mortality indicates that an increase in malaria transmission measures results in more malaria deaths which in turn inflate overall mortality. The stronger effect of prevalence on malaria specific mortality is because there is a clear biological cause and effect [33] and malaria infection can and does lead to mortality, however, the relationship between prevalence and all-cause mortality is diluted by other causes of mortality.

From these findings, it is worth noting that prevalence as a measure of transmission shows more stability in determining mortality over longer periods of time (4–5 years) compared to annual measures. Comparing estimates of the relation between mortality and malaria transmission measured by prevalence (of parasitaemia and confirmed malaria) in the current study, incidence measured as slide positivity rate (SPR) [10] and the log of EIR from capture better the relationship between malaria transmission and mortality (Table 4). Confirmed malaria prevalence averaged over 4–5 years is likely to be more stable in areas of high transmission and therefore a useful measure of transmission over a long period while incidence and EIR capture the malaria-mortality relationship better over shorter periods [10, 34]. These differences could be due to the fact that PP one-off estimates can be misleading indicators of long-term transmission potential, since they vary markedly with season [35]. These short-term fluctuation would then make it harder to associate yearly PP measures with mortality occurring all year round; suggesting that population based prevalence surveys do represent long term transmissions as opposed to short term changes.

**Table 4:** Comparison of estimates measuring the relation between malaria transmission and mortality from previous studies and current work

	Transmission measure	Neonates	Infants	Child	Older child	Adults	Elderly	All ages
(All-cause)	Log EIR^a^	*3.91* (*3.53–4.32*)	*3.64* (*3.40–3.89*)	*4.29* (*3.89–4.73*)	–	–	–	–
	Slide Positivity Rate (Incidence)^b^	0.89 (0.13–5.70)	3.10 (0.36–13.12)	*4.29* (*2.78–13.29*)	0.48 (0.15–2.05)	0.73 (0.39–1.42)	1.70 (0.79–4.45)	*1.55* (*1.04–2.80*)
	Annual parasite prevalence^c^	1.23 (0.71–2.10)	1.04 (0.75–1.42)	1.11 (0.80–1.52)	1.15 (0.67–1.98)	*1.23* (*1.01–1.50*)	1.04 (0.86–1.28)	1.10 (0.97–1.25)
	Annual clinical malaria prevalence^c^	1.03 (0.44–2.32)	1.22 (0.68–1.84)	1.38 (0.87–2.19)	1.97 (0.88–4.17)	1.16 (0.86–1.58)	0.99 (0.72–1.36)	1.16 (0.97–1.40)
	Five-year parasite prevalence^d^	0.64 (0.29–1.34)	1.32 (0.86–2.04)	1.50 (0.98–2.23)	1.38 (0.69–2.69)	1.23 (0.94–1.57)	1.04 (0.78–1.35)	1.15 (0.96–1.37)
	Five-year clinical malaria prevalence^d^	1.05 (0.30–3.51)	1.65 (0.81–3.26)	*1.89* (*1.00–3.51*)	*3.94* (*1.34–11.1*)	0.99 (0.64–1.54)	1.07 (0.69–1.66)	*1.32* (*1.01–1.74*)
(Malaria-specific)	Log EIR^a^	–	*4.35* (*3.72–4.95*)	*4.29* (*3.61–5.06*)	–	–	–	–
	Slide Positivity Rate (Incidence)^b^	–	1.36 (0.23–9.85)	*9.48* (*5.11–37.94*)	0.02 (0.003–0.33)	0.27 (0.02–3.24)	0.59 (0.01–13.15)	1.37 (0.51–3.73)
	Annual parasite prevalence^c^	–	1.31 (0.71–2.37)	1.50 (0.92–2.41)	0.80 (0.32–2.06)	0.78 (0.32–1.80)	*3.42* (*1.39–8.63*)	1.31 (0.95–1.78)
	Annual clinical malaria prevalence^c^	–	0.94 (0.34–2.47)	1.58 (0.73–3.23)	3.65 (0.94–12.79)	0.36 (0.08–1.49)	1.67 (0.38–6.52)	1.34 (0.82–2.14)
	Five-year parasite prevalence^d^	–	1.73 (0.74–4.21)	*2.29* (*1.17–4.29*)	0.56 (0.14–2.03)	1.55 (0.48–4.86)	2.24 (0.67–7.44)	*1.66* (*1.07–2.54*)
	Five-year clinical malaria prevalence^d^	–	2.23 (0.55–8.36)	*3.37* (*1.23–8.93*)	*7.56* (*1.20–39.5*)	0.60 (0.08–3.91)	0.77 (0.09–5.64)	*2.50* (*1.27–4.81*)

Estimates are Bayesian posterior medians and 95% Bayesian Credible Intervals (BCI)

Statistical important effects are indicated in italic

Source: ^a^Amek et al. [34]; ^b^Khagayi et al. [10]

^c^Annually aggregated data. Current work

^d^Data aggregated over 4–5 years. Current work

Higher socioeconomic status, shorter distance to health facilities and increasing altitude are known protective factors that were statistically important for both, all-cause and malaria-specific mortality. Individuals at a higher social status are more likely to live in well-constructed houses that offer better protection against endophagic/endophilic malaria vectors that transmit malaria in sub-Saharan Africa, afford better nutrition and pay for superior treatment [36]. Increasing elevation is associated with lower temperatures which increase the development time of both vector and parasite [37], resulting in lower transmission. Similarly, it has been shown that distance to health facilities influences mortality [38].

Lack of association between net use and mortality across ages except for yearly data among the elderly could be due to data aggregation at village level which diminished the expected individual level protection associated with net use reported in earlier studies during the 90′s and early 2000′s in the same region [34, 39]. This change from earlier years could have been due to a number of factors among them ITN’s having achieved maximum benefits, compromised effectiveness due to misuse/pyrethroid resistance or other unmeasured factors which countered their protective effect (Hamel et al. pers.commun.). The diminished effect of net use might also be due to use of self-reported net use information which could lead to bias as it does not measure constant use. The negative effect of net use on malaria-specific mortality among the elderly, a group that has not been well researched in malaria cannot be explained adequately, and requires further investigation. However, it could be hypothesized that since mortality is generally high in this age-group, at the same time society considers them vulnerable, issuance and use of ITNs could be higher and hence their protective effect is masked.

There are inherent limitations in survey data and in estimating malaria mortality using verbal autopsy that could influence the study results. First, the surveys were conducted in specific months (i.e. in April, just before the rains (or just as they were starting) or June/July after the rains were ending.); therefore, the prevalence estimates of may be biased by unexpected changes in climatic and environmental factors in other. Use of verbal autopsy as a tool for determining cause of death has been criticized [30], even though recent improvements in the InterVA coding have been said to reduce classification errors, especially at population level [40]. Despite these limitations, the 9-year data in the study have been collected consistently in the same area using rigorous data collection methods and strict quality control measures. These data are thus unique in studying the relation between malaria prevalence and mortality across all groups in this population within a high endemic area.

## Conclusion

From yearly cross-sectional malaria prevalence surveys, the study showed that; (i) Clinical malaria at population level best captures the association with mortality among children aged 1–4 years and 5–14 year olds. It can also be used as a marker of malaria mortality in the general population. (ii) Prevalence as a measure of transmission is more stable over longer periods of time (4 to 5 years) compared to incidence or EIR which better capture the malaria-mortality relationship on a yearly basis. However, its lower size effect compared to clinical malaria may underestimate malaria deaths. Analyses of data from other HDSS sites or similar platforms with differing levels of malaria endemicity different socio-economic status, or different access to effective anti-malarial drugs would be useful in understanding the contribution of parasite prevalence to mortality across age groups.

## Additional files

**Additional file 1.** Study designs for the malaria survey data during 2007-2015.

**Additional file 2.** Bayesian model formulation.

**Additional file 3.** Posterior estimates of the effects of prevalence on all-cause mortality aggregated annually.

**Additional file 4.** Posterior estimates of the effects of prevalence on malaria-specific mortality aggregated annually.

## Data Availability

Data were obtained with permission of the Kisumu HDSS and Malaria branch steering committee. Any data requests may be sent to the respective steering committees, through Dr. Simon Kariuki (Skariuki@kemricdc.org) or Dr. Stephen Munga (Smunga@kemri.org).

## Abbreviations

ACT: artemisinin-based combination therapy
AIDS: acquired immune deficiency syndrome
BCI: Bayesian Credible Interval
CDC: Centers for Disease Control and Prevention
CI: Credible Intervals
HDSS: Health and Demographic Surveillance Systems
EIR: entomological inoculation rate
HIV: human immunodeficiency virus
KEMRI: Kenya Medical Research Institute
LLIN: long lasting insecticide-treated nets
MCA: multiple correspondence analysis
MCMC: Markov Chain Monte Carlo
MTIMBA: Malaria Transmission Intensity and Mortality Burden across Africa
PP: parasite prevalence
PYO: person years of observation
RR: rate ratios
SES: socio-economic status
SPR: slide positivity rate
SSA: Sub-Saharan Africa
VA: verbal autopsy
