# Supplementary material for: Modelling the relationship between malaria prevalence as a measure of transmission and mortality across age groups
Source: Malar J. 2019 Jul 23;18:247. doi: 10.1186/s12936-019-2869-9 (PMC6651924; doi:10.1186/s12936-019-2869-9)
Supplement: Supplementary file 1 — Additional file 1. Study designs for the malaria survey data during 2007-2015. [file 12936_2019_2869_MOESM1_ESM.docx]

**Additional file 1:**

*Study designs for the malaria survey data during 2007-2015*

| **Month/Year of Study** | **Area(s)** | **Design and Context** | **Sampling design** | **Sample Size** |
| --- | --- | --- | --- | --- |
| April 2007 | Whole HDSS area | HDSS | Systematic Random Sampling | 1270 |
| April 2008 | Whole HDSS area | HDSS | Systematic random sampling | 1039 |
| April 2009 | Whole HDSS area | HDSS | Cluster randomization (cluster unit = village) Villages selected by random sampling proportional to size | 2508 |
| April 2010 | Whole HDSS area | HDSS | Systematic random sampling | 5243 |
| June-July 2011 | Whole HDSS area | HDSS | Systematic random sampling | 2091 |
| June-July 2012 | Whole HDSS area | HDSS | Systematic random sampling | 2719 |
| June-July 2013 | Whole HDSS area | HDSS | Systematic random sampling | 2358 |
| 2014 | Whole HDSS area | HDSS | Systematic random sampling | 1934 |
| 2015 | Whole HDSS area | HDSS | Systematic random sampling | 1756 |
