# Supplementary material for: Modelling the relationship between malaria prevalence as a measure of transmission and mortality across age groups
Source: Malar J. 2019 Jul 23;18:247. doi: 10.1186/s12936-019-2869-9 (PMC6651924; doi:10.1186/s12936-019-2869-9)
Supplement: Supplementary file 2 — Additional file 2. Bayesian model formulation. [file 12936_2019_2869_MOESM2_ESM.docx]

**Additional file 2:**

*Bayesian model formulation*

Let Y*_jt_* be the observed number of deaths (all-cause or malaria-specific) in village *j* at time *t, j=1,2,…n* and *t=1,2,…9* for the model fitted on the annual data. We assume that Y*_jt_* arises from a negative binomial distribution$, Y_{jt}\sim NB(\mu_{jt},r\boldsymbol{)}$ where $\mu_{jt}$, is the expected number of deaths and *r* is the dispersion parameter. We modeled malaria prevalence and other covariates (***X***) by on the log scale of $\mu_{jt}$

$log\left( \mu_{jt} \right)=log\left( N_{jt} \right)+\beta_{0}+{\sum_{1}^{k} \beta_{k}X}_{jtk}+\phi_{j}$ , *k=1,2,…K*

where $N_{jt}$ the total person time contributed by persons in village *j* at time *t* (in person years, py), ${\boldsymbol{\beta}=(\beta_{1},\beta_{2},\ldots,\beta_{K})}^{T}$ are the regression coefficients (malaria prevalence and other predictors) and $\phi_{j}$ the village specific spatial effects. We assumed that ${\boldsymbol{\phi}=(\phi_{1},\phi_{2},\ldots\phi_{n})}^{T}$ are modeled by a Gaussian process, that is $\boldsymbol{\phi} \sim MVN(0,\sigma_{1}^{2}R)$ and that $R$ is an exponential correlation matrix of the distance between villages, i.e. $R_{ij}=exp(-\rho d_{kl})$ where $d_{kl}$ is the distance between villages $k$ and $l$, $\rho$ is the rate of *correlation* decay with distance. The minimum distance at which the spatial correlation is less than 5% is called effective range and is defined by the value of $3/\rho$ (1). The $\sigma_{1}^{2}$ is the variance of the spatial process. We specified non-informative normal prior distributions with mean zero and large variance for the regression coefficients $\beta_{i}\sim N(0, {10}^{3})$, an inverse gamma prior for $\sigma_{1}^{2}$, that is $\sigma_{1}^{2} \sim IG(2.01, 1.01)$ and a Uniform prior distribution for $\rho$, that is $\rho\sim U(a, b)$, where $a$ and $b$ are chosen such as the effective range is within the maximum and minimum distances of the village’s locations.

The Bayesian models were then fitted using Markov Chain Monte Carlo (MCMC) simulation algorithm in OpenBugs version 3.1.2 (Imperial College and Medical Council, London, UK) to estimate model parameters (2). We ran a two chain sampler for 100,000 iterations, discarding the first 10,000 iterations. Convergence was assessed by the Gelman-Rubin diagnostic (3).

*References*

1. Ecker MD, Gelfand AE. Bayesian Variogram Modeling for an Isotropic Spatial Process. J Agric Biol Environ Stat. 1997;2(4):347–69.

2. Gelfand AE, Smith AFM. Sampling-Based Approaches to Calculating Marginal Densities. J Am Stat Assoc. 1990 Jun 1;85(410):398–409.

3. Gelman A, Rubin DB. Inference from Iterative Simulation Using Multiple Sequences. Stat Sci. 1992 Nov;7(4):457–72.
