# Supplementary material for: Modelling the relationship between malaria prevalence as a measure of transmission and mortality across age groups
Source: Malar J. 2019 Jul 23;18:247. doi: 10.1186/s12936-019-2869-9 (PMC6651924; doi:10.1186/s12936-019-2869-9)
Supplement: Supplementary file 4 — Additional file 4. Posterior estimates of the effects of prevalence on malaria-specific mortality aggregated annually. [file 12936_2019_2869_MOESM4_ESM.docx]

**Posterior estimates of the effects of prevalence on malaria-specific mortality aggregated annually**

|  | **Infants** | **1-4 yrs.** | **5-14 yrs.** | **15-59 yrs.** | **60 plus** | **Overall**** |
| --- | --- | --- | --- | --- | --- | --- |
| **Covariate** | **RR** | **RR** | **RR** | **RR** | **RR** | **RR** |
|  | **(95% BCI*)** | **(95% BCI)** | **(95% BCI)** | **(95% BCI)** | **(95% BCI)** | **(95% BCI)** |
| PP*** | 1.31 | 1.50 | 0.8 | 0.78 | 3.42 | 1.31 |
|  | (0.71-2.37) | (0.92-2.41) | (0.32-2.06) | (0.32-1.80) | (1.39-8.63) | (0.95-1.78) |
| Clinical malaria*** | 0.94 | 1.58 | 3.65 | 0.36 | 1.67 | 1.34 |
|  | (0.34-2.47) | (0.73-3.23) | (0.94-12.79) | (0.08-1.49) | (0.38-6.52) | (0.82-2.14) |
| Net use | 1.08 | 0.94 | 0.64 | 0.75 | 2.05 | 0.97 |
|  | (0.68-1.65) | (0.67-1.31) | (0.33-1.27) | (0.40-1.42) | (1.04-4.34) | (0.79-1.21) |
| Distance to facility |  |  |  |  |  |  |
| 0 – 1 km | 1 | 1 | 1 | 1 | 1 | 1 |
| 1 – 2 km | 1.13 | 0.93 | 1.23 | 1.38 | 1.04 | 1.06 |
|  | (0.86-1.50) | (0.76-1.15) | (0.78-1.98) | (0.94-2.07) | (0.67-1.63) | (0.92-1.22) |
| >2 km | 1.16 | 1.03 | 1.61 | 1.14 | 1.11 | 1.11 |
|  | (0.86-1.57) | (0.82-1.30) | (1.00-2.65) | (0.74-1.79) | (0.68-1.81) | (0.94-1.22) |
| SES |  |  |  |  |  |  |
| Poorest | 1 | 1 | 1 | 1 | 1 | 1 |
| Poor | 0.89 | 0.94 | 0.84 | 0.81 | 0.98 | 0.89 |
|  | (0.67-1.19) | (0.75-1.16) | (0.55-1.28) | (0.55-1.19) | (0.63-1.53) | (0.78-1.03) |
| Least poor | 0.87 | 0.81 | 0.71 | 0.77 | 1.18 | 0.84 |
|  | (0.64-1.18) | (0.64-1.03) | (0.45-1.13) | (0.51-1.15) | (0.74-1.88) | (0.72-0.98) |
| Year 2007 | 1 | 1 | 1 | 1 | 1 | 1 |
| 2008 | 3.05 | 3.37 | 2.09 | 1.36 | 2.44 | 2.74 |
|  | (1.94-4.94) | (2.32-5.02) | (0.71-6.82) | (0.73-2.57) | (1.01-6.23) | (2.14-3.48) |
| 2009 | 1.93 | 2.37 | 4.76 | 2.06 | 1.35 | 2.17 |
|  | (1.11-3.38) | (1.54-3.69) | (1.74-15.02) | (1.07-3.96) | (0.47-3.84) | (1.66-2.86) |
| 2010 | 1.72 | 1.69 | 3.72 | 0.9 | 2.52 | 1.66 |
|  | (1.11-2.76) | (1.18-2.48) | (1.56-10.63) | (0.52-1.62) | (1.17-5.82) | (1.32-2.11) |
| 2011 | 1.3 | 1.35 | 1.82 | 0.73 | 1.12 | 1.14 |
|  | (0.78-2.24) | (0.91-2.06) | (0.65-5.75) | (0.37-1.43) | (0.45-2.84) | (0.88-1.48) |
| 2012 | 1.15 | 1.08 | 3.07 | 0.67 | 1.13 | 1.05 |
|  | (0.70-1.94) | (0.72-1.63) | (1.22-9.07) | (0.36-1.27) | (0.47-2.87) | (0.81-1.35) |
| 2013 | 1.34 | 1.3 | 2.72 | 0.97 | 1.81 | 1.25 |
|  | (0.83-2.22) | (0.88-1.94) | (1.09-7.86) | (0.56-1.77) | (0.82-4.33) | (0.99-1.60) |
| 2014 | 0.78 | 0.82 | 1.57 | 0.79 | 1.08 | 0.79 |
|  | (0.39-1.52) | (0.48-1.38) | (0.47-5.40) | (0.37-1.63) | (0.39-3.05) | (0.57-1.08) |
| 2015 | 0.19 | 0.51 | 1.24 | 0.16 | 0.41 | 0.36 |
|  | (0.01-0.56) | (0.27-0.93) | (0.35-4.37) | (0.03-0.50) | (0.05-1.53) | (0.23-0.54) |
| Elevation |  |  |  |  |  |  |
| 1147 – 1243 | 1 | 1 | 1 | 1 | 1 | 1 |
| 1244 – 1293 | 1.07 | 1.19 | 0.89 | 1.05 | 0.9 | 1.1 |
|  | (0.76-1.50) | (0.88-1.61) | (0.51-1.53) | (0.62-1.77) | (0.49-1.63) | (0.88-1.37) |
| 1294 – 1327 | 0.49 | 1.23 | 0.97 | 0.81 | 1.05 | 0.9 |
|  | (0.32-0.75) | (0.88-1.68) | (0.55-1.72) | (0.47-1.41) | (0.55-1.98) | (0.69-1.17) |
| 1328 – 1365 | 0.72 | 1.13 | 0.98 | 0.96 | 1.12 | 0.95 |
|  | (0.47-1.11) | (0.78-1.58) | (0.51-1.86) | (0.54-1.71) | (0.56-2.25) | (0.72-1.23) |
| >1365 | 0.4 | 0.94 | 1.28 | 0.61 | 1.21 | 0.75 |
|  | (0.23-0.66) | (0.62-1.38) | (0.64-2.54) | (0.31-1.19) | (0.56-2.61) | (0.55-1.00) |
| Spatial Variance | 0.19 | 0.16 | 0.06 | 0.14 | 0.22 | 0.22 |
|  | (0.04-0.36) | (0.04-0.36) | (0.03-0.29) | (0.03-0.36) | (0.05-0.37) | (0.05-0.37) |
| Range^$^ | 15.91 | 18.22 | 50.42 | 20.75 | 13.44 | 13.36 |
|  | (8.23-74.54) | (8.25-84.10) | (10.35-97.07) | (8.39-90.56) | (8.18-60.19) | (8.17-63.63) |

The effects are presented as the median of mortality rate ratios (RR) and 95% Bayesian credible intervals (BCI) adjusted for geographical variation and other predictors

^**^ Age-adjusted

^***^ They are obtained from different models. Estimates of the rest of the predictors are from the models with confirmed malaria and do not differ from the PP model. PP estimates are only provided for comparison purposes

^$^ Minimum distance in kilometers at which spatial correlation is less than 5%
